# Specific Effects of Chronic Dietary Exposure to Chlorpyrifos on Brain Gene Expression—A Mouse Study

**DOI:** 10.3390/ijms18112467

**Published:** 2017-11-20

**Authors:** Maria Michela Pallotta, Raffaele Ronca, Rosa Carotenuto, Immacolata Porreca, Mimmo Turano, Concetta Ambrosino, Teresa Capriglione

**Affiliations:** 1 Dipartimento di Biologia, Università di Napoli Federico II, Via Cinthia 21, 80126 Napoli, Italy; mariamichela.pallotta@unina.it (M.M.P.); rafronca@unina.it (R.R.); rosa.carotenuto@unina.it (R.C.); mimmo.turano@unina.it (M.T.); 2 IRGS, Biogem, Via Camporeale, Ariano Irpino, 83031 Avellino, Italy; ip9@sanger.ac.uk (I.P.); concetta.ambrosino@unisannio.it (C.A.); 3 Dipartimento di Scienze e Tecnologie, Università del Sannio, Via Port’Arsa 11, 82100 Benevento, Italy

**Keywords:** chlorpyrifos, organophosphate insecticides, *Mus musculus*, developmental neurotoxicity, Parkinson’s disease, neurodegenerative diseases, PCR-array

## Abstract

Chlorpyrifos (CPF) is an organophosphate insecticide used to control pests on a variety of food and feed crops. In mammals, maternal exposure to CPF has been reported to induce cerebral cortex thinning, alteration of long-term brain cognitive function, and Parkinson-like symptoms, but the mechanisms of these processes are not fully understood. In this study, we aimed to gain a deeper understanding of the alterations induced in the brains of mice chronically exposed to CPF by dietary intake. For our purpose, we analysed F1 offspring (sacrificed at 3 and 8 months) of *Mus musculus*, treated in utero and postnatally with 3 different doses of CPF (0.1-1-10 mg/kg/day). Using RT^2^ Profiler PCR Arrays, we evaluated the alterations in the expression of 84 genes associated with neurodegenerative diseases. In the brains of exposed mice, we evidenced a clear dose–response relationship for AChE inhibition and alterations of gene expression. Some of the genes that were steadily down-regulated, such as *Pink1*, *Park 2*, *Sv2b*, *Gabbr2*, *Sept5* and *Atxn2*, were directly related to Parkinson’s onset. Our experimental results shed light on the possibility that long-term CPF exposure may exert membrane signalling alterations which make brain cells more susceptible to develop neurodegenerative diseases.

## 1. Introduction

Developmental exposure to OP (organophosphate) insecticides has been suggested to have long-lasting negative impacts, including decreased motor skills and cognitive abilities, increased signs of attention deficit/hyperactivity disorder (ADHD), and also altered brain morphology [1,2,3,4,5].

Despite the restrictions on their use, human exposure to OPs pesticides through food or the use of products containing chemicals is frequent. This is particularly harmful to children, who eat and breathe more in relation to their body weight. Consequently, the same exposure results in higher concentrations of pesticides in their bodies than in the adults [6].

A report published in January 2015 by the Swedish Environmental Research Institute [7] clearly showed that some pesticides are commonly absorbed into the body through food, such as the widely used OP insecticide chlorpyrifos, the levels of which were measured by the concentration of its metabolite TCP (3,5,6-trichloro pyridine-2-phenol).

Chlorpyrifos (CPF) is, among the organophosphorus insecticides, one of the most commonly employed in the cultivation of fruit and vegetables. In a study of pesticide residues in urine samples from 128 women conducted in Skåne, Sweden in 2010, TCP was found in all samples and in higher concentrations in vegetarians than in non-vegetarians [7].

Although a number of studies carried out both in vivo and in vitro have ratified the neurotoxic effects of CPF—even at low concentrations—and have suggested its association with the neurodegenerative diseases, the compound is still used, because it controls a broad spectrum of insects in both the agricultural and human settings [8,9,10]. In June 2000, the EPA banned almost all household uses of CPF, and in 2006 it limited the variety of crops on which it can be applied, with the final aim being to revoke all chlorpyrifos permissions on 30 October 2015, but the final decision was postponed to 31 March 2017.

The main mechanism of CPF action is similar to that of the other organophosphates: inhibition of acetylcholinesterase (AChE), which results in accumulation of the acetylcholine and subsequent hyperactivity in the cholinergic system. However, multiple developmental studies on animal models have reported that chronic CPF exposure can alter brain development and neuronal morphogenesis even in the absence of significant AChE inhibition [11,12,13,14,15,16,17]. Research data on the CPF neurotoxic effects in young animals are of particular interest, because the pesticide seems to elicit alterations in the levels of genes promoting neural differentiation. Betancourt et al. [18], found that the expression of two factors critical to brain development, NGF (Neural growth factor), and RLN (Reelin), was significantly reduced in the brains of neonatal rats that are exposed to both low and high CPF concentrations. NGF and Reelin protein are known to be important for the establishment of normal neuronal processes and synaptic plasticity. Furthermore, Reelin triggers nerve cells to migrate to their proper locations.

A comparative histological study of the brain has highlighted the effects of CPF on the cleavage plane orientation of neural progenitors in the cerebral cortex; exposure to the pesticide causes unnatural horizontal mitotic figures, associated with cell shrinkage and apoptosis [19]. Moreover, a growing number of the studies have associated increased Parkinson’s disease (PD) risk with insecticide exposure, especially chlorpyrifos [20].

Recently, several genes associated with familial PD have been shown to be sensitive targets of environmental factors, and both genetic and environmental factors are believed to contribute to the development of sporadic PD [21]. Notably, chlorpyrifos is among the pesticides associated with greater SNCA (α-Synuclein) expression, a protein critically involved in Parkinson’s, in cell line models of dopaminergic neurons [13,21,22]. In addition, a significant reduction of the dopaminergic neurons at 16d and 46d were evidenced in the CPF-postnatally treated rats, suggesting that the exposure of CPF may induce dopaminergic neuronal injury [23].

The present study aimed to evaluate the effects of a chronical exposure to the environmental detected concentrations of CPF in mice exposed in utero and postnatally to the pesticide. The pregnant females were fed for all the pregnancy and the nursing period [24] with three different concentrations of CPF (0.1 mg/kg/day, 1 mg/kg/day, 10 mg/kg/day), that are commonly found in fruits and vegetables. Afterwards, weaned mice were fed in the same manner until the time of sacrifice. Mice of the exposed and control groups were sacrificed at 3 and 8 months, corresponding respectively to the young-adult and adult. Analysis was performed to evaluate the alterations in the expression level of genes known to be involved in neurodegeneration.

## 2. Results

**Figure 1:**
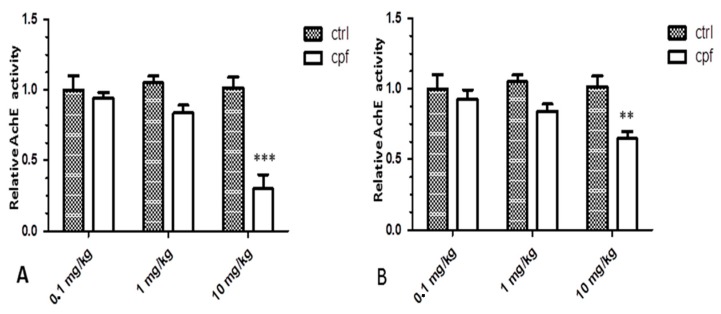
Differences of AchE activity in brains of mice treated with three CPF concentrations (0.1-1-10 mg/kg/day), sacrificed at 3 (**A**) and 8 (**B**) months. ** *p* < 0.01, and *** *p* < 0.001.

No significant brain ChE inhibition was seen following 0.1–1 mg/kg CPF exposure in any of the mice analyzed at 3 and 8 months, whereas a reduction in brain cholinesterase activity was reported only at the highest dosage (10 mg/kg CPF) at both stages (80–30% inhibition, respectively) (Figure 1).

### 2.1. PCR Array and Data Validation by qRT-PCR

Using 84 gene plates, we assayed the expression of genes directly or potentially involved in Parkinson’s as well as in other neurodegenerative diseases. The threshold of gene expression to select genes altered by CPF exposure was 2.0-fold. We selected and analyzed all the genes that exceeded the threshold value. 

**Figure 2:**
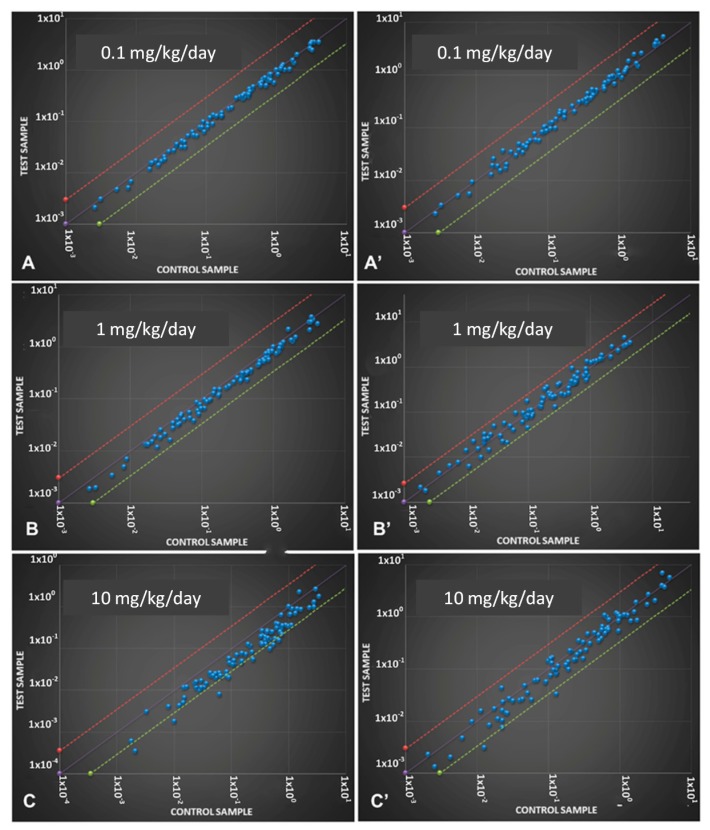
Scatter plots of all up-regulated and down-regulated genes involved in Parkonson’s disease (PD) that were investigated in 3 ((**A**) 0.1 mg/kg/day; (**B**) 1 mg/kg/day; (**C**) 10 mg/kg/day) and 8 ((**A’**) 0.1 mg/kg/day; (**B’**) 1 mg/kg/day; (**C’**) 10 mg/kg/day) month-old mice exposed to different CPF dosages. The orange line indicates a fold change (2^ΔΔ*C*t^) of 1. The other two lines indicate a two-fold change in the gene expression threshold.

At three months, qRT-PCR-array analysis did not show any significant gene alteration at 0.1 mg/kg/day, a slight gene down-regulation was present at 1 mg/kg/day, while a general down-regulation of the transcript levels was present for most of the genes at 10 mg/kg/day (Figure 2A–C). 

The greatest number of deregulated genes were associated with the highest dosage (10 mg/kg CPF). Based on the cut-off criteria of (2^−Δ*C*t^) > 2, *p*-value < 0.05 we screened 48 differently expressed transcripts (DETs), as reported in Table 1. 

The majority of the down-regulated transcripts belonged to proteins involved in synaptic transmission and plasticity, gabaergic and dopaminergic signaling.

The only exception to the decreasing trend was represented by the *Ubiquitin C* (*UBC*) gene, whose level of transcripts, albeit within the threshold values, increased gradually (from 0.35 to 0.50) with increasing CPF concentration, as shown by the three graphs reporting the values at the three concentrations (Figure 2A–C).

#### 2.1.1. qRT-PCR Validation at Three Months

A subset consisting of six genes (*Park2*, *Pink1*, *SV2B*, *SEPT5*, *GABBR2* and *UBC*), deregulated at 10 mg/kg/day and involved in different networks, was sampled for verification using real-time PCR. Results from RT-PCR generally agreed with the microarray analysis, although the absolute degree of change could differ between the methods. 

**Figure 3:**
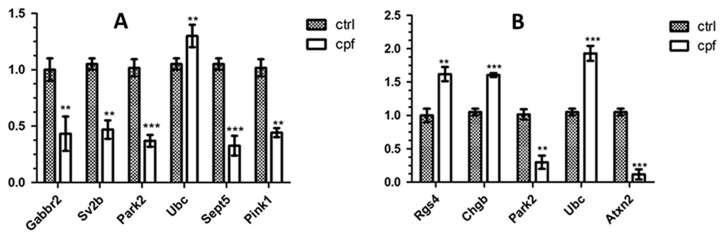
Real-time PCR analysis of genes involved in PD in 3-month- (**A**) and 8-month- (**B**) old mice. Data are presented as mean with SD. Statistical significance was determined using *t*-tests with Holm-Sidak correction for multiple comparison, ** *p* < 0.01, and *** *p* < 0.001.

*UBC* confirmed the increasing level of the transcript, +0.30, at the higher concentration when compared to the control group. The expression of *Pink1*, *Sept5*, *Park2*, *Gabbr2* and *Sv2b* was instead down-regulated, ranging from −0.5 to −0.3 (Figure 3A).

**Figure 4:**
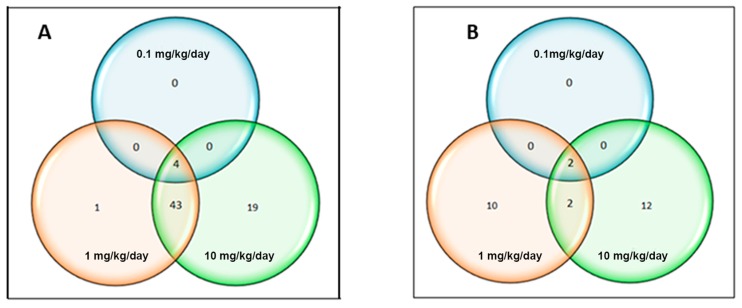
Venn diagram showing the differentially expressed genes across all the dosages (0.1, 1, 10 mg/kg/day) in 3-month- (**A**) and 8-month- (**B**) old mice. This diagram illustrates the overlap of mouse brain gene expression changes (*p* < 0.05) across dosages following CPF exposure.

The Venn diagram (Figure 4A) shows overlap of gene expression changes with the three CPF dosing conditions in three-month-old mice. Only genes whose range of variation from the control was <0.5 or >0.5, and whose *p*-values < 0.05, were chosen for the analysis.

As shown in Figure 4A, there was relatively little overlap among deregulated genes across all three dosages. Only four genes were deregulated at all three concentrations. Two of these down-regulated genes are attributed to dopaminergic signaling, *Park2* and *Nr4a2*, one to GABAergic signaling, *Gabbr2*, and the last one, *Sv2b,* is involved in transmembrane transport activity (Table 2). 

#### 2.1.2. PCR Array and qRT-PCR Validation at Eight Months

The landscape of gene alterations after the CPF exposure appeared quite different in the 8-month-old mice. Again, no genes beyond the threshold values were reported at the lowest CP concentrations, 0.1 × 10^1^ mg/kg/day; while at 10 mg/kg/day, there was a general recovery of the values of gene transcripts reported as deregulated at 3 months. Nevertheless, *Park2*, *Ataxin 2*, *DRD2* and *UBC* values were still altered (Figure 2A’–C’). 

Applying the same criteria used for analyzing the three-month-old mice, we reported only 14 differently expressed transcripts (DETs) (Table 3). 

In particular, at 8 months, down-regulated transcripts were significantly enriched for proteins involved in dopaminergic signaling, whereas the transcripts of genes involved in ubiquitination, parkin complex and cell signaling were reported as up-regulated.

A subset of five genes (*Park2*, *Atxn2*, *Rgs4*, *Chgb* and *UBC*), deregulated at 10 mg/kg/day, involved in the different networks was sampled for verification using real-time PCR. Results from RT-PCR (Figure 3B) confirmed microarray analysis. 

qRT-PCR validated the results reporting a decrease for *Park2* (−0.30) and *Atxn2* (−0.10), and increases of +0.66 for *Rgs4*, +1.64 for *Chgb,* and +1.96 for *Ubc*, which reports a further increase of the transcript level (from +1.30 at 3 months, to +1.96 at 8 months) (Figure 3B).

Also at 8 months, Venn diagram analysis was performed on the genes, whose range of variation compared to the control group was >0.5 and <0.5, *p*-value < 0.05 (Figure 4B). In this range of values, only two genes were commonly deregulated at the three concentrations, both showing increasing levels of transcripts: *Ubc* and *Casp9* (Table 4). 

## 3. Discussion

In this study, an analysis was made of the putative changes in the expression of PD-related genes in the brains of 3- and 8-month-old mouse groups exposed chronically, in utero and postnatally, to three different CPF concentrations (0.1, 1, 10 mg/kg/day). The results showed direct evidence that long-lasting exposure to CPF targets the expression of the several genes known to be related to the emergence of PD and other neurodegenerative disorders in humans [13].

Initially, we evidenced a clear dose–response relationship in the brains of 3- and 8-month-old mice exposed to the pesticide both for AChE inhibition and for alterations of gene expression, although the panel of altered genes differed between the two age groups.

Overall, exposed chlorpyrifos produced a mixed pattern of up- and down-regulation, which is more evident in the groups treated with a higher CPF concentration (10 mg/kg/day).

However, while there was still a dose–response relationship, appreciable worsening was not detectable with increased exposure time; rather, the brains of the eldest mice showed a general recovery of gene functionality with ageing.

The total number of gene networks affected sharply reduced at 8 months, and AchE values shifted from highly inhibited—approximately 80% in 3-month-old mice—to 30% in the eldest mice.

Physiological cholinergic neuronal activity is important to correctly modulate the expression of genes involved in proliferation, differentiation and in the apoptotic events necessary to shape the embryo’s different brain regions. In addition, it is known that cholinergic circuit control is important for correct postnatal neurogenesis [25].

Altered production of AchE has been shown to affect the regulation of critical genes involved in neurogenesis, such as the NGF (Neurotrophin nerve growth factor) and several neuropeptides [18,26]. Furthermore, in adult rats, immunohistochemistry studies have demonstrated that Ach levels, reduced by exposure at 10 mg/kg/day subcutaneous injection of CPF for 21 days, resulted in the up-regulation of CRHBP (corticotropin releasing hormone binding protein), and NPY (neuropeptide Y) transcription in the CA1 (*Cornu Ammonis*) region of the hippocampus [26].

However, Zhang et al. [27], studying the effect of AchE alterations on Parkinson’s disease using acetylcholinesterase-deficient mice, found that these mice have reduced dopaminergic neuron loss and lower expression levels of apoptotic proteins. They conclude that a deficiency or inhibition of acetylcholinesterase can decrease apoptosis and protect dopaminergic neurons in the neurotoxin model of Parkinson’s [27]. 

In the 3-month-old mice, our data confirm this trend; in utero and postnatally, long-lasting CPF exposure dramatically alters ACh levels in the brains of mice, putatively interfering with the signaling of the genes enrolled in PD that we analyzed. The genes most affected are those regulating synaptic transmission and ubiquitination, therefore impairing GABA (Gamma-aminobutyric acid), and DOPA (Dopamine) neurotransmitter pathways.

In particular, the transcript levels of the GABBR2 receptor, one of the master genes in GABA signaling, are clearly reduced in the 10 mg/kg/day CPF-treated group. This is of importance because GABA_B_ receptors play a crucial role in maintaining excitatory/inhibitory balance in the brain synapsis. Recently, in a paper of 2016, Błaszczyk formulated the “GABA collapse” hypothesis, in which the GABA decline is proposed to play a prominent role in the development and progression of PD and other neurodegenerative diseases [28,29,30,31].

As reported by several studies, individuals with autism can show reduced levels of GAB(B) receptors in the cingulate cortex and fusiform gyrus. GABBR2, in particular, was significantly reduced in the cerebellum of these patients [32]. 

Similarly, we could consider the reduction of several transcripts involved in synaptic functionality, such as *Sv2b*, *Lrrk2*, *Sept5*, *Syngr3*, *Syt1*, *Nsf* and *Nsg1.* These all play a role in synaptic transmission, modulating correct maintenance and release of neurotransmitter vesicles. Alteration of the endo- and exocytotic mechanism, and the impairment of intracellular trafficking, which are recurrent in both patients of Parkinson’s and experimental models of the disease [33]. This specificity may result from the particular anatomy of dopaminergic neurons (DA) neurons. DA cells have an enormous axonal field, with the number of synaptic terminals far exceeding the number of neurons [34], and loss of DA nerve terminals seems to precede DA cell body loss in PD [35]. Possibly, the morphology of these neurons makes them rely more on local SV cycling, and mechanisms that affect these processes make this system more vulnerable. 

These putative changes in neurotransmitters may impact and impair the functionality of the three-month-old mice’s DA cells, in particular due to the peculiar morphology of these neurons, which have a number of synaptic terminals greatly exceeding the number of cells [34,35]. Four of the currently known genes involved in DOPA signaling, *park2*, *pink1*, *DRD2* and *slc6a3*, whose monogenic mutations are found in early or juvenile onset PD patients [36]—were decreased in the 10 mg/kg/day group of 3-month-old mice, while synuclein levels were not altered, as happens in Parkinsonians. Some PD genes, such as *Parkin* and *Pink1*, share roles in mitochondrial functionality. *Parkin* and *Pink1*, together with *Park7*, regulate the autophagic degradation of damaged mitochondria. Inactivating mutations in these genes cause autosomal recessively inherited PD. 

In the general landscape of down-regulation found at three months, the rising level of Ubc is even more interesting. *Ubiquitin C* (*UbC*) has been described as the most responsive gene to cellular stress, even though little is known about the molecular mechanisms modulating its expression [37]. The Ubc1 homologue was found to be highly induced in the brains of patients with Alzheimer’s disease, and was upregulated in neuronal cells after exposure to the amyloid-β peptide. Moreover, in a recent transcriptome analysis, upregulated levels of UBC transcripts were reported in the cerebrospinal fluid of PD patients and were considered, together with other deregulated transcripts, as potential PD diagnosis and treatment RNA biomarkers [38]. Here, we suggest that, in the 3- to 8-month-old mice, the impaired transcripts of these genes might temporarily mimic a condition of early-onset of Parkinson’s.

At eight months, the general putative recovery of the down-regulated transcripts correlates with the decrease of the brain cholinesterase inhibition (approximately 30%). We hypothesize that this might result from the better detoxifying capacity of the eldest mice, as reported by several studies. Individual differences in detoxification capacities for specific organophosphorus (OP) compounds have been reported to be largely due to either the differences in catalytic efficiency or the abundance of the high-density lipoprotein (HDL)-associated enzyme paraoxonase (PON1). Studies on rats of different ages highlighted increased PON1 activity in adults, which may justify the variation between 3- and 8-month-old mice [39,40]. Contrastingly, recent studies have shown that, after repeated CPF treatment, the mice seemed to develop some tolerance to CPF-induced effects, suggesting that the detoxifying mechanisms are possibly involved in the induction of tolerance. Compensatory mechanisms were not active in thyroids of mice treated with the same CPF concentrations [24]. 

Anyway, our data does not allow the exclusion of the hypothesis that other unknown esterases, besides acetylcholinesterase, might be potential targets of CPF, as has also been suggested previously [9,17]. Neverthless, the general improvement in the number of gene transcripts back within the threshold of variability in the 8-month-old mice does not apply to *ATXN2* (a *Park2* substrate) and *DRD2*, whose transcript levels instead remain low. We hypothesize that, although the apparent general recovery in the transcript numbers of the Parkinson’s genes studied, a certain cellular alteration is still conceivable.

In particular, low levels of *DRD2* in the 8-month-old mice treated with 10 mg/kg/day, seem to confirm a standing altered functionality of the DOPA cells. In addition, putative problems in neuronal communication are confirmed by the increasing levels of the transcripts of CHGB and RGS4, two proteins that are implicated in the onset of several neurodegenerative disorders. RGS4 is a negative regulator of G protein signaling expressed in the nervous system [41]. Usually regulated by the dopaminergic agents, RGS4 is up-regulated in PD patients in whom the DOPA level decreases [41]. RGS4 up-regulation, together with a DRD2 reduction, in transcript numbers seems to be further evidence of DOPA-signaling alteration. Interestingly, recent studies have suggested direct RGS4 inhibition as a new target for Parkinson’s care [42,43]. 

Decreased levels of CHGB-derived peptides have been discovered in the cerebrospinal fluid of multiple sclerosis (MS) patients by Mo et al., who showed that, within a model of multiple sclerosis for mice, CGB levels were elevated in the neurons prior to the onset of MS symptoms. Additionally, they suggested that the initial elevation of CHGB, prior to symptom onset, is due to inflammatory processes. It is conceivable to hypothesize that long-lasting CPF exposure at 8 months gives rise to a similar state. 

With the exception of *Park2* (whose down-regulation level does not vary with ageing), the genes involved in the Ubiquitination Pathway, *UBC* and *Ube2k*, return to their physiological values (or become higher in a number of RNA transcripts) in the 8-month-old mice. Interestingly, *UBC* and *Ube2k* values almost progressively doubled compared to 3 months, mimicking what happens during PD progression [38].

Increased levels of *Park7* and *HSPA4*, listed in the Parkin complex, may, instead, be explained as an adaptive response to the chronic stress induced by exposure to the pesticide. Both genes are considered neuroprotectors, and we hypothesize that, during aging, higher expression of *Park7* and *HSPA4* might have been required by neurons to stabilize *Pink1* and protect cells from oxidative stress and apoptosis [44,45]. Moreover, the *HSPA4* protein, a member of the HSP70 family, has a role in the degradation of misfolded proteins. 

The findings at 8 months shed light on a possible mechanism by which CPF, at environmental doses, may exert a long-term cell signaling alteration that may induce neurodegenerative disorders with aging. 

Data present in the literature suggest different reasons for the behavioral alterations after CPF exposure. Williams et al. (2014) [46] performed an analysis of rodent model studies involving CPF/CPO exposure and concluded that gestational and/or perinatal CPF exposure is not likely to be associated with the development of autism-like behaviors in humans. Contrastingly, spatial learning impairment was shown in prepubertal guinea pigs prenatally exposed to a single chlorpyrifos dose [47]. In this case, while acute AChE inhibition was not displayed, neurotoxic effects such as persistent aberrant behavior and cognitive function in adults, as well as altered levels of neuroproteins in the developing brain, were reported. Other studies have suggested different ways, including oxidative stress, by which CPF exposure could be linked to behavioral alterations [48,49].

Because the data are contrasting and examine only shorter exposure periods or different experimental design, further histological studies comparing protein expression levels in dopaminergic neurons, as well as behavioral observations would help to assess the role of CPF in neurodegenerative disease development and will be the focus of future studies.

## 4. Materials and Methods

### 4.1. Animals and Treatments

Animal experiments (Progetto MODO 6 February 2015) were performed in compliance with the European Council Directive 86/609/EEC and the Italian Legislation on Animal Experimentation (D.Lvo 116/92), and the procedures were approved (ID number 21-2009) by the Ethical Committee named CESA (Committee for the Ethics of the Experimentations on Animals) of the Biogem Institute of Genetics Research “Gaetano Salvatore” (IRGS). The project was communicated to the employee office of the Ministry of Health following the rules of the D.Lvo 116/92.

Mice were kept under standard facility conditions (22 ± 2 °C, 55 + 10% humidity, 12:12 h light-dark cycle) in a specific pathogen-free facility. Animals kept under standard facility conditions received water and standard diet (4RF21 form Mucedola, Settimo Milanese, Italy) ad libitum. The mice were fed for the entire duration of the pregnancy and lactation with a contaminate feed to which was added clorpyrifos at different concentrations, 44, 4.4 and 0.44 mg/kg (Mucedola) to allow dosages of 10 mg/kg/day, 1 mg/kg/day, 0.1 mg/kg/day respectively. A group of mice without treatment was used as control. CD1 dams (outbred strain, 8 mice/treatment group) were exposed, 7 days before the mating, to pesticides dosed at 10 mg/kg/day, 1 mg/kg/day, 0.1 mg/kg/day, and the combinations of higher and lower doses, administrating CPF by food at 44, 4.4 and 0.44 mg/kg (Mucedola) until weaning. Therefore, the offspring were exposed through the mothers from gestational day 0 (GD0) until weaning. Then, at weaning, the offspring were divided into groups of 10 mice, and subjected to the same diet as their parents until the time of sacrifice. Animals were sacrificed by carbon dioxide inhalation at 3 and 8 months in order to observe the effects over the time of the administration of the pesticide. The brains were rapidly collected, and transferred into RNAlater^®^ Solution (Invitrogen, Carlsbad, CA, USA) for molecular analysis.

### 4.2. Protein Extraction and AchE Assay

After removal, three brains of each group were homogenized with 600 μL of lysis buffer containing PBS, 0.5% NP-40 and protease inhibitors, 1 mM PMSF (Roche, Basilea, Switzerland). Thirty micrograms of proteins were used for assessing AchE activity. 4 μL of DTNB (5,5′-dithiobis-(2-nitrobenzoic acid), dissolved in 10 mM absolute ethanol, brought to the final volume of 200 μL with 0.2 M phosphate buffer pH 7.4, were added to each sample.

Optical densities were measured at a wavelength of 412 nm. The enzymatic reaction was quantified against a blank without substrate. The AChE assay had three replicates for each sample. Activity data were analyzed using the one-way analysis of variance followed by the Tukey-Kramer test and expressed as mean ± standard error. The differences between the treatments and controls were tested (*p* < 0.05).

### 4.3. RNA Isolation and cDNA

Total RNA was extracted from the brains of three mice of each experimental group, according to the TRI-Reagent protocol (Sigma Aldrich, Saint Louis, MO, USA). RNA quality was assessed using the 2100 Bioanalizer (Agilent Technologies, Palo Alto, CA, USA). First-strand cDNA, used for all amplification reactions, was synthesized from 2 μg of pooled RNAs from three brains of each group of mice using the RT2 First Strand Kit (QIAGEN, Hilden, Germany) following the manufacturer’s instructions. 

### 4.4. Quantification Assay (PCR-Array)

qPCR was performed using ready-to-use mouse Parkinson’s disease RT^2^ Profiler PCR Array (QIAGEN, Hilden, Germany) containing primers for 84 tested (Table S1) and 5 housekeeping genes, and controls for RT and PCR reactions. The whole volume of cDNA synthesized was used for the preparation of reaction mixture. To each 96-well plate, 25 μL reaction mixture based on RT^2^ SYBR Green qPCR Mastermix (QIAGEN, Hilden, Germany), was added. Thermal cycling was performed as recommended by plate manufacturers for IQ5 (10 min initial denaturation at 95 °C followed by 40 cycles: 15 s in 95 °C, with 1 min amplification in 60 °C). All plates had positive PCR controls and reverse transcription controls. The calculations of contamination with mouse genomic DNA were performed according to the manufacturer’s instructions, and showed the presence of genomic DNA in an acceptable range, not influencing the experiment performance. Values of cycle threshold (*C*_t_) obtained in quantification were used for the calculations of fold changes in mRNA abundance accordingly to 2^−ΔΔ^*^C^*^t^ method. β2 microglobulin was chosen from the group of five housekeeping genes as the best. Changes in the mRNA level of the evaluated genes were assessed in all groups in relation to the control group of animals with mRNA abundance set up arbitrarily as 1.

### 4.5. Data Analysis

Data were expressed as fold change. Fold change (2^−ΔΔ*C*t^ method) is the normalized gene expression (2^−Δ*C*t^) in the test sample divided by the normalized gene expression (2^−Δ*C*t^) in the control sample. The differences between the experimental groups and the control group were analyzed by Student’s *t*-test and used for comparisons with RT^2^ Profiler PCR Array data analysis software version 3.5 (SABiosciences, Frederick, MD, USA). *p* < 0.05 was considered to be statistically significant.

### 4.6. Validation by qRT-PCR

The first-strand cDNA, used for all quantitative validation reactions, was synthesized from 2 μg of the pooled RNAs previously used for PCR-array, using SuperScript III Reverse Transcriptase (Invitrogen, Carlsbad, CA, USA) and used for the validation.

Primers used for qRT_PCR validation were designed using software Primer 3Plus (http://www.bioinformatics.nl/cgi-bin/primer3plus/primer3plus.cgi/) on sequences found in Genbank (Table 5).

Real-time PCR was performed using Power SYBER Green Master Mix Kits (Invitrogen) using the 96-well optical reaction plate in 20 µL total reaction volume.

For transcript relative quantification, samples were normalized to β2 microglobulin as a housekeeping control to account for possible differences in the quantity and quality of the cDNA used in the experiments. PCR was carried out with the following thermal profile: stage 1—95 °C for 3 min; Stage 2—40 cycles of 95 °C for 15 s and 60 °C for 45 s; Stage 3—95 °C for 15 s, 60 °C for 1 min and 95 °C for 15 s; Stage 4—dissociation curve with 95 °C for 15 s, 60 °C for 1 min and 95 °C for 15 s. A separate dissociation curve assay was performed for each reaction to confirm gene-specific amplification. Reactions were conducted on an Applied Biosystem 7500 (Frederick) Real-Time PCR System. Statistical significance was determined using a *t*-test analysis with the Holm-Sidak correction for the multiple comparison methods using a GraphPad Prism 6 software (Hilden, Germany).

## 5. Conclusions

Our findings suggest that dopaminergic neurotransmission and mitochondrial integrity are the main targets of CPF and both can be considered as sensitive biomarkers of exposure, contributing to the overall spectrum of neurotoxicity. 

## Figures and Tables

**Table 1:** Relative fold change and functions of DETS in 3-month-old mice.

Gene Name	Functions	Fold Change
*CASP9*	Apoptosis	−0.35
*MAPK9*	Apoptosis	−0.39
*NR4A2*	Apoptosis, Dopaminergic Signaling	−0.34
*CASP3*	Apoptosis, Mitochondrial	−0.44
*OPA1*	Apoptosis, Mitochondrial	−0.29
*CDH8*	Cell Adhesion Molecules	−0.35
*FN1*	Cell Adhesion Molecules	−0.38
*NFASC*	Cell Adhesion Molecules	−0.33
*NRXN3*	Cell Adhesion Molecules	−0.35
*TPBG*	Cell Adhesion Molecules	−0.16
*PTEN*	Cell Adhesion Molecules, Apoptosis, Mitochondrial	−1.44
*DRD2*	Dopaminergic Signaling	−0.35
*SLC6A3*	Dopaminergic Signaling	−0.34
*GABBR2*	GABAergic Signaling	−0.13
*ATP2B2*	Ion Transport	−0.39
*CXXC1*	Ion Transport	−0.41
*EGLN1*	Ion Transport	−0.40
*GRIA3*	Ion Transport	−0.40
*HTR2A*	Ion Transport	−0.36
*KCNJ6*	Ion Transport	−0.26
*NSF*	Ion Transport	−0.29
*SLIT1*	Ion Transport	−0.34
*SRSF7*	Ion Transport	−0.41
*ALDH1A1*	Other Parkinson’s Disease Genes	−0.37
*BASP1*	Other Parkinson’s Disease Genes	−0.21
*CHGB*	Other Parkinson’s Disease Genes	−0.40
*NCOA1*	Other Parkinson’s Disease Genes	−0.40
*RTN1*	Other Parkinson’s Disease Genes	−0.34
*STUB1*	Parkin Complex, Ubiquitination	−0.32
*ATXN2*	Parkin Substrate	−0.32
*ATXN3*	Parkin Substrate	−0.33
*GPR37*	Parkin Substrate	−0.30
*SYT11*	Parkin Substrate, Synaptic Vesicles	−0.35
*SV2B*	Synaptic Vesicles	−0.22
*SYNGR3*	Synaptic Vesicles	−0.22
*SYT1*	Synaptic Vesicles	−0.32
*SEPT5*	Synaptic Vesicles, Dopaminergic Signaling	−0.23
*UBC*	Ubiquitination	+0.49
*USP34*	Ubiquitination	−0.36
*LRRK2*	Ubiquitination, Mitochondrial, Synaptic Vesicles	−0.39
*PARK2*	Ubiquitination, Mitochondrial, Dopaminergic Signaling	−0.18
*PINK1*	Ubiquitination, Mitochondrial, Dopaminergic Signaling	−0.23

**Table 2:** Venn diagram summary for the three-month-old mice.

Chlorpyrifos Concentrations	DETs (Differently Expressed Transcripts)	Altered Genes
0.1 mg/kg/day	0	0
0.1–1 mg/kg/day	0	0
0.1–10 mg/kg/day	0	0
1–10 mg/kg/day	43	*Ubc*, *Aldh1a1*, *Apc*, *Atp2b2*, *Atxn2*, *Atxn3*, *Basp1*, *Bdnf*, *Casp3*, *Casp9*, *Cdc27*, *Cdh8*, *Chgb*, *Cxxc1*, *Drd2*, *Egln1*, *Fbxo9*, *Fn1*, *Gabbr2*, *Gpr37*, *Htr2a*, *Kcnj6*, *Lrrk2*, *Ncoa1*, *Nefl*, *Nfasc*, *Nrxn3*, *Nsf*, *Nsg1*, *Ntrk2*, *Opa1*, *Pan2*, *Park2*, *Pink1*, *Pten*, *Rgs4*, *Sept5*, *Srsf7*, *Sv2b*, *Syngr3*, *Syt1*, *Ube2k*, *Usp34*
1 mg/kg/day	1	Gbe1
10 mg/kg/day	19	*App*, *Cadps*, *Casp7*, *Ddc*, *Dlk1*, *Gria3*, *Hspa4*, *Park7*, *Psen2*, *Rtn1*, *Skp1a*, *Slc6a3*, *Slit1*, *Stub1*, *Syt11*, *Tpbg*, *Uch11*, *Ywhaz*
0.1-1-10 mg/kg/day	4	*Park2*, *Nr4a2*, *Gabbr2*, *Sv2b*

**Table 3:** Relative fold change and functions of DETS in 8-month-old mice.

Gene Name	Functions	Fold Change
*CASP1*	Apoptosis	+1.13
*CASP9*	Apoptosis	+0.55
*PSEN2*	Apoptosis	+0.59
*DRD2*	Dopaminergic Signaling	−0.38
*CHGB*	MAP Kinase Signaling	+0.67
*RGS4*	MAP Kinase Signaling	+0.95
*HSPA4*	Parkin Complex, Mitochondrial	+0.45
*PARK7*	Parkin Complex, Mitochondrial, Dopaminergic Signaling	+0.44
*ATXN2*	Parkin Substrate	−0.29
*CDC27*	Ubiquitination	+0.57
*FBXO9*	Ubiquitination	+0.45
*UBC*	Ubiquitination	+0.89
*UBE2K*	Ubiquitination	+0.63
*PARK2*	Ubiquitination, Mitochondrial, Dopaminergic Signaling	−0.28

**Table 4:** Venn diagram summary of the eight-month-old mice.

Chlorpyrifos Concentrations	DETs	Altered Genes
0.1 mg/kg/day	0	
0.1–1 mg/kg/day	0	
0.1–10 mg/kg/day	0	
1–10 mg/kg/day	2	*Ube2k*, *Park2*
1 mg/kg/day	10	*Bdnf*, *Cul2*, *Gabbr2*, *Gbe1*, *Pan2*, *Ppid*, *Snca*, *Dlk1*, *Kcnj6*, *Uchl1*
10 mg/kg/day	12	*Cdc27*, *Chgb*, *Fbxo9*, *Hspa4*, *Park7*, *Psen2*, *Rgs4*, *Ube2l3*, *Atxn2*, *Ddc*, *Drd2*, *Fn1*
0.1-1-10 mg/kg/day	2	*Ubc*, *Casp9*

**Table 5:** Oligo sequences used for qRT-PCR.

Oligo Name	Sequence 5′–3′
Gabbr FOR	TCCGGAACGGGGAAAGAATG
Gabbr REV	TCCGACCCCTGGAACCTTAT
Park2 FOR	ACCCACCTACAACAGCTTTTTC
Park2 REV	CAGCAAGATGGGCCCTGG
Sept5 FOR	GACCCCAGAGGACAAACAGG
Sept5 REV	ACCATGAGCGTGAAGTCGAA
Sv2b FOR	TGCTGGAGATGGGCAAACAT
Sv2b REV	TGAACACCTTTTCCGGGGTC
Atxn FOR	CCCGGGCGTACAACCTTTAT
Atxn REV	TGTCGCTGTTGGGGCATATT
Ubc FOR	GCCCAGTGTTACCACCAAGA
Ubc REV	CCCCATCACACCCAAGAACA
Chgb FOR	CTCACCAGGAGGCAAACGAT
Chgb REV	AGTTCCAGATCCATCGCAGC
Rgs4 FOR	GCCAGAGGGTAAGCCAAGAA
Rgs4 REV	TCCTCGCTGTATTCCGACTTC
Pink1 FOR	CTGCCTGAGATGCCTGAGTC
Pink1 REV	GTGCAGACGGTCTCTTGCT

## Supplementary Materials

Supplementary materials can be found at www.mdpi.com/1422-0067/18/11/2467/s1.

## Abbreviations

CPF	Chlorpyrifos
DETs	Differently expressed transcripts
PON1	Paraoxonase
