# Supplementary material for: Specific Effects of Chronic Dietary Exposure to Chlorpyrifos on Brain Gene Expression—A Mouse Study
Source: Int J Mol Sci. 2017 Nov 20;18(11):2467. doi: 10.3390/ijms18112467 (PMC5713433; doi:10.3390/ijms18112467)
Supplement: Supplementary file 1 [file ijms-18-02467-s001.pdf]

Gene table: RT² Profiler PCR Array

UniGene	GenBank	Symbol	Description
Mm.250866	NM_013467	Aldh1a1	Aldehyde dehydrogenase family 1, subfamily A1
Mm.384171	NM_007462	Apc	Adenomatosis polyposis coli
Mm.277585	NM_007471	App	Amyloid beta (A4) precursor protein
Mm.321755	NM_009723	Atp2b2	ATPase, Ca++ transporting, plasma membrane 2
Mm.260900	NM_009125	Atxn2	Ataxin 2
Mm.485508	NM_029705	Atxn3	Ataxin 3
Mm.29586	NM_027395	Basp1	Brain abundant, membrane attached signal protein 1
Mm.1442	NM_007540	Bdnf	Brain derived neurotrophic factor
Mm.260881	NM_012061	Cadps	Ca2+-dependent secretion activator
Mm.1051	NM_009807	Casp1	Caspase 1
Mm.34405	NM_009810	Casp3	Caspase 3
Mm.35687	NM_007611	Casp7	Caspase 7
Mm.336851	NM_009812	Casp8	Caspase 8
Mm.88829	NM_015733	Casp9	Caspase 9
Mm.89845	NM_145436	Cdc27	Cell division cycle 27 homolog (S. cerevisiae)
Mm.1022	NM_009861	Cdc42	Cell division cycle 42 homolog (S. cerevisiae)
Mm.441131	NM_007667	Cdh8	Cadherin 8
Mm.255241	NM_007694	Chgb	Chromogranin B
Mm.291707	NM_029402	Cul2	Cullin 2
Mm.17537	NM_028868	Cxxc1	CXXC finger 1 (PHD domain)
Mm.12906	NM_016672	Ddc	Dopa decarboxylase
Mm.157069	NM_010052	Dlk1	Delta-like 1 homolog (Drosophila)
Mm.41970	NM_010077	Drd2	Dopamine receptor D2
Mm.140619	NM_053207	Egln1	EGL nine homolog 1 (C. elegans)
Mm.28584	NM_023605	Fbxo9	F-box protein 9
Mm.7995	NM_010200	Fgf13	Fibroblast growth factor 13
Mm.193099	NM_010233	Fn1	Fibronectin 1
Mm.101909	NM_001081141	Gabbr2	Gamma-aminobutyric acid (GABA) B receptor, 2
Mm.396102	NM_028803	Gbe1	Glucan (1,4-alpha-), branching enzyme 1
Mm.409670	NM_010338	Gpr37	G protein-coupled receptor 37
Mm.327681	NM_016886	Gria3	Glutamate receptor, ionotropic, AMPA3 (alpha 3)
Mm.239865	NM_008300	Hspa4	Heat shock protein 4
Mm.214351	NM_172812	Htr2a	5-hydroxytryptamine (serotonin) receptor 2A
Mm.328720	NM_010606	Kcnj6	Potassium inwardly-rectifying channel, subfamily J, member 6
Mm.37558	NM_025730	Lrrk2	Leucine-rich repeat kinase 2
Mm.68933	NM_016961	Mapk9	Mitogen-activated protein kinase 9
Mm.1287	NM_010838	Mapt	Microtubule-associated protein tau
Mm.301039	NM_010881	Ncoa1	Nuclear receptor coactivator 1
Mm.1956	NM_010910	Nefl	Neurofilament, light polypeptide
Mm.326702	NM_182716	Nfasc	Neurofascin
Mm.3507	NM_013613	Nr4a2	Nuclear receptor subfamily 4, group A, member 2
Mm.425766	NM_172544	Nrxn3	Neurexin III
Mm.260117	NM_008740	Nsf	N-ethylmaleimide sensitive fusion protein
Mm.7414	NM_010942	Nsg1	Neuron specific gene family member 1
Mm.130054	NM_008745	Ntrk2	Neurotrophic tyrosine kinase, receptor, type 2

UniGene	GenBank	Symbol	Description
Mm.274285	NM_133752	Opa1	Optic atrophy 1 homolog (human)
Mm.244183	NM_133992	Pan2	PAN2 polyA specific ribonuclease subunit homolog (S. cerevisiae)
Mm.311110	NM_016694	Park2	Parkinson disease (autosomal recessive, juvenile) 2, parkin
Mm.277349	NM_020569	Park7	Parkinson disease (autosomal recessive, early onset) 7
Mm.18539	NM_026880	Pink1	PTEN induced putative kinase 1
Mm.295252	NM_026352	Ppid	Peptidylprolyl isomerase D (cyclophilin D)
Mm.347009	NM_011563	Prdx2	Peroxiredoxin 2
Mm.330850	NM_011183	Psen2	Presenilin 2
Mm.245395	NM_008960	Pten	Phosphatase and tensin homolog
Mm.41642	NM_009062	Rgs4	Regulator of G-protein signaling 4
Mm.221275	NM_153457	Rtn1	Reticulon 1
Mm.235998	NM_009115	S100b	S100 protein, beta polypeptide, neural
Mm.20365	NM_213614	Sept5	Septin 5
Mm.42944	NM_011543	Skp1a	S-phase kinase-associated protein 1A
Mm.353923	NM_172523	Slc18a2	Solute carrier family 18 (vesicular monoamine), member 2
Mm.16228	NM_007450	Slc25a4	Solute carrier family 25 (mitochondrial carrier, adenine nucleotide translocator), member 4
Mm.41993	NM_010020	Slc6a3	Solute carrier family 6 (neurotransmitter transporter, dopamine), member 3
Mm.40322	NM_015748	Slit1	Slit homolog 1 (Drosophila)
Mm.17484	NM_009221	Snca	Synuclein, alpha
Mm.299906	NM_019763	Spen	SPEN homolog, transcriptional regulator (Drosophila)
Mm.292016	NM_146083	Srsf7	Serine/arginine-rich splicing factor 7
Mm.277599	NM_019719	Stub1	STIP1 homology and U-Box containing protein 1
Mm.273082	NM_153579	Sv2b	Synaptic vesicle glycoprotein 2 b
Mm.26032	NM_011522	Syngr3	Synaptogyrin 3
Mm.289702	NM_009306	Syt1	Synaptotagmin I
Mm.379376	NM_018804	Syt11	Synaptotagmin XI
Mm.139815	NM_009333	Tcf7l2	Transcription factor 7-like 2, T-cell specific, HMG-box
Mm.1292	NM_009377	Th	Tyrosine hydroxylase
Mm.20864	NM_011627	Tpbg	Trophoblast glycoprotein
Mm.1104	NM_009457	Uba1	Ubiquitin-like modifier activating enzyme 1
Mm.331	NM_019639	Ubc	Ubiquitin C
Mm.240044	NM_011665	Ube2i	Ubiquitin-conjugating enzyme E2I
Mm.319512	NM_016786	Ube2k	Ubiquitin-conjugating enzyme E2K (UBC1 homolog, yeast)
Mm.3074	NM_009456	Ube2l3	Ubiquitin-conjugating enzyme E2L 3
Mm.29807	NM_011670	Uchl1	Ubiquitin carboxy-terminal hydrolase L1
Mm.119155	NM_001190401	Usp34	Ubiquitin specific peptidase 34
Mm.32321	NM_009496	Vamp1	Vesicle-associated membrane protein 1
Mm.227704	NM_011696	Vdac3	Voltage-dependent anion channel 3
Mm.3360	NM_011740	Ywhaz	Tyrosine 3-monooxygenase/tryptophan 5-monooxygenase activation protein, zeta polypeptide
Mm.328431	NM_007393	Actb	Actin, beta
Mm.163	NM_009735	B2m	Beta-2 microglobulin
Mm.343110	NM_008084	Gapdh	Glyceraldehyde-3-phosphate dehydrogenase
Mm.3317	NM_010368	Gusb	Glucuronidase, beta
Mm.2180	NM_008302	Hsp90ab1	Heat shock protein 90 alpha (cytosolic), class B member 1
